# Forkhead box O3 transcription factor fine-tunes IL-6 expression in mycobacteria-infected macrophages

**DOI:** 10.3389/fimmu.2026.1888327

**Published:** 2026-08-17

**Authors:** Manel Mejri, Yoldoz Bouzguenda, Sonia Ben Hamouda, Khadija Essafi-Benkhadir, Makram Essafi

**Affiliations:** 1 Institut Pasteur de Tunis, Laboratory of Transmission, Control and Immunobiology of Infections LTCII LR16IPT02, Tunis, Tunisia; 2 Université Tunis El Manar, Tunis, Tunisia; 3 Institut Pasteur de Tunis, Laboratoire d’Epidémiologie Moléculaire et Pathologie Expérimentale LR16IPT04, Tunis, Tunisia

**Keywords:** tuberculosis, immune response, macrophage, M1/Th1, IL-6, FOXO3

## Abstract

**Introduction:**

Tuberculosis (TB) remains the deadliest infectious disease in humans; however, a robust immune response can either protect against or control the infection. Household contacts of TB patients can remain uninfected for decades, while a quarter of the world's population that is latently infected can remain healthy for years. Characterization of the effectors of such an immune response would help in developing novel host-directed tools to better treat and/or prevent TB. IL-6 is a crucial cytokine that plays a central role in orchestrating a protective TB immune response; however, a delicate balance between its beneficial and detrimental effects must be maintained to effectively control the infection. Here, we aimed to characterize the cellular effectors that regulate IL-6 expression in mycobacteria-infected cells.

**Methods:**

We used PI3K and AKT inhibitors, as well as FOXO3-specific siRNA, to modulate FOXO3 activity in BCG-infected macrophages and assessed the effect on IL-6 expression. Reporter gene assays were performed using four IL-6 promoter constructs (-600 bp, -900 bp, -1.5 kb, and -2 kb). *In silico* analysis was carried out using Genomatix software and the Eukaryotic Promoter Database to identify Forkhead binding motifs, followed by mutagenesis experiments to validate their function.

**Results:**

The PI3K/AKT/FOXO3 axis regulates IL-6 expression in human macrophages: FOXO3 activation (via PI3K or AKT inhibition) induced IL-6 expression, while FOXO3 silencing reduced IL-6 production in BCG-infected macrophages. FOXO3 positively regulated IL-6 promoter activity across all cloned fragments, with the strongest effect at -1.5 kb; extension to -2 kb modulated this FOXO3-mediated induction. *In silico* analysis identified three FOXO3-specific binding sites (-786, -1331, and -1631). Mutagenesis identified the motif at -1331 as the major positive regulator of IL-6 expression, while the motif at -1631 mediated the negative regulation exerted by the distal promoter fragment.

**Discussion:**

We report for the first time that FOXO3 exerts both positive and negative regulation of IL-6 transcription, fine-tuning its expression in mycobacteria-infected macrophages. Together with our previous report on FOXO3-mediated suppression of IL-10, these results highlight the crucial role of FOXO3 in the TB immune response and support targeting the PI3K/Akt/FOXO3 axis as a host-directed approach to improve TB treatment and prevention.

## Introduction

*Mycobacterium tuberculosis* (*Mtb*), the causative agent of tuberculosis (TB), is the most common infectious killer of humans, with an estimated 1.4 million deaths and 10.4 million new cases worldwide (1). The emergence of drug-resistant *Mtb* strains (2), the limited protection conferred by the sole *Mycobacterium bovis*, bacille Calmette et Guerin (BCG) vaccine against pulmonary TB (3), and the advent of acquired immune deficiency syndrome (AIDS) are the three major causes of the high global burden of TB (4). The fact that an estimated quarter of the human population is controlling their latent *Mtb* infection indicates that an effective host immune response can mostly restrain the spread of TB (5). Moreover, household contacts of patients with TB can remain uninfected for decades, indicating the ability of a robust immune system to confer protection against TB (6).

Uncovering the clues behind such an immune response would be of great benefit for the development of host-directed tools to better treat and/or prevent TB.

The immune response to *Mtb* is a multifaceted process in which cytokines are involved at every stage, determining the fate of infection to either a detrimental or a protective state of the host (7). The long co-evolutionary history between humans and *Mtb* has resulted in the bacterium developing sophisticated mechanisms to manipulate the host immune response, often by modulating cytokine production and signaling, to ensure its survival and transmission (8). This intricate interplay underscores the importance of a nuanced understanding of cytokine networks in TB for developing more effective host-directed therapies and vaccines. The IL-6 has been shown to contribute to early host defense mechanisms against TB (9). It can potentiate initial immune responses following *Mtb* infection by contributing to the recruitment of immune cells to the site of infection and influencing the local inflammatory milieu (10, 11). Some studies suggest that IL-6 is important for controlling high-dose *Mtb* infection where a rapid and robust immune response is critical (12, 13). Furthermore, IL-6 plays a significant role in shaping the adaptative immune response and is one of the key cytokines that drives the differentiation of naive CD4+ T cells into T helper 17 (Th17) cells (14). Additionally, IL-6 is a major inducer of the acute phase response in the liver, leading to the production of inflammatory markers like C-reactive protein (CRP) and serum amyloid A (SAA), which are often elevated during active TB (15).

Despite these protective contributions, the role of IL-6 in TB is not uniformly beneficial, and its effects are nuanced. Although IL-6 is involved in Th17 differentiation, an overzealous Th17 response or excessive neutrophil recruitment driven by IL-6-related pathways can sometimes contribute to immunopathology and tissue damage in the lungs (13). The balance between the pro-inflammatory and immunoregulatory effects of IL-6 can be influenced by the timing of its expression, its concentration, and its interaction with other cytokines in the complex network governing the immune response to *Mtb*.

IL-10 is an effective modulator of the immune response during infection that prevents excessive tissue damage, which can be exploited by *Mtb* to promote its persistence within the host (16). High levels of IL-10 have often been associated with more severe forms of TB, increased bacterial burden, and impaired *Mtb* clearance in both human studies and animal models. *Mtb* itself can actively induce IL-10 production from host cells as an immune evasion strategy (17).

Overall, the timing, location, and magnitude of IL-6 and IL-10 expression are critical determinants of their overall impact on the outcome of *Mtb* infection. An appropriate level of IL-10 is necessary to prevent immunopathology, but excessive IL-10 can compromise the host’s ability to control the infection. On the other hand, IL-6 plays a crucial role in TB effective immune response but its excessive expression is detrimental for the host (12).

Uncovering the cellular signaling pathways and their target transcription factors controlling IL-6 and IL-10 expression may then help understanding the mechanisms by which these cytokines contribute to several aspects of anti-TB immunity. This would also help developing strategies that uncouple the beneficial effects of these two cytokines from their detrimental ones on anti-mycobacterial immunity.

The expression of both IL-6 and IL-10 is regulated by the canonical Phosphoinositide 3-Kinase (PI3K)/AKT pathway (18–20). We have previously shown that Forkhead box O3 (FOXO3) transcription factor, which is negatively regulated by Akt, inhibits IL-10 secretion in mycobacteria-infected macrophages through the direct binding to and repression of IL-10 promoter (21). It is worth noting that in this latter work, knocking down of FOXO3 also led to the decrease of IL-6 transcription, suggesting a positive regulation exerted by FOXO3 on IL-6 expression. Moreover, activation of FOXO3 by an AKT inhibitor during BCG vaccination led to the suppression of IL-10, a more robust M1/Th1 immune response, and enhanced the protective efficacy of the BCG vaccine in mice and guinea pig (22). IL-6 is one of the major cytokines driving the Th1 immune response. However, we did not explore the potential direct regulation of IL-6 expression by FOXO3.

Based on our data along with the fact that the expression of IL-10 and IL-6 is closely related, we suggest that the PI3K/AKT/FOXO3 axis may also regulate the level of IL-6. In this study, we aimed to check such hypothesis. We show that FOXO3 binds to several regions of the IL-6 promoter, finely regulating IL-6 expression in BCG-infected macrophages. This indicates that, besides IL-10, the PI3K/AKT/FOXO3 axis regulates the expression of IL-6, strengthening or modulating the M1/Th1 immune response phenotype. Overall, the current study, along with our previous works on the role of FOXO3 in TB immune response, suggests FOXO3 as a potential target to develop host-directed tools to boost the immune response during TB treatment or to enhance the efficacy of vaccination.

## Materials and methods

### Cell culture

The human monocytic THP1 cell line (ATCC TIB-202; American Type Culture Collection, Manassas, VA, USA) was cultured in RPMI-1460 medium (Thermo Fisher Scientific, Waltham, MA, USA) as described previously (21, 23). The media was supplemented with 10% heat inactivated fetal bovine serum (FBS; Thermo Fisher Scientific, Waltham, MA, USA), 12 mM HEPES buffer, 0.1 mM non-essential amino-acids, 1 mM sodium pyruvate, and 1% penicillin/streptomycin (Life Technologies, Carlsbad, CA, USA). Cells were maintained at 37 °C in a humidified 5% CO2 atmosphere and passage every 2–3 days to maintain exponential growth. For differentiation into macrophages, THP1-derived macrophages (TDMs), THP1 monocytes were seeded at 5×10^5^ cells/ml in 24 well-plates and treated with 20 ng/ml phorbol-myristate 13 acetate (PMA; Sigma Aldrich, St. Louis, MS, USA) for 48h, followed by a 24h recovery in RPMI-free medium prior to infection or transfection experiments. Differentiated THP1 macrophages were infected with *Mycobacterium bovis* BCG (Pasteur strain 1173P2) at a multiplicity of infection (MOI) of 1:10 for 2 hours in order to assure the infection of all cells and translocation of FOXO3 to the nucleus (23). Thereafter, extracellular bacilli were removed by washing twice with PBS, and cells were incubated in fresh medium containing inhibitors of AKT for the indicated time points. The drugs were used at the indicated concentrations: 5 µM of MK-2206 (MK) 5 µM of AKT inhibitor (AKTi) or 3 µM of Miltefosine (Mil), all from Selleckchem, USA. Supernatants were collected 24h post-infection and stored at -20 °C for subsequent cytokine assays, while cells were harvested for protein extraction for western blotting.

Human Embryonic Kidney 239-cells (HEK 293; ATCC, Manassas, VA, USA) were maintained in Dulbecco’s Modified Eagle’s medium (DMEM, GIBCO, Invitrogen, Carlsbad, CA, USA) supplemented with 10% FBS under standard culture conditions (37 °C, 5% CO2). Cells were routinely passaged at 70-80% confluence after dissociation with 0.25% Trypsin-EDTA. For transfection experiments, cells were treated as described previously (24), with slight modifications. Briefly, cells were seeded at 2×10^5^ cells per well in 24 well plates and transfected the following day with plasmid DNA using Lipofectamine™ 2000 transfection reagent (Thermo Fisher Scientific, Waltham, MA, USA), following the manufacturer’s protocol. Cells were harvested 24h post transfection for subsequent assays.

### Colony-forming unit assay

The CFU assay was performed as detailed in (21), with slight modifications. Following differentiation of THP-1 cells into TDMs, or after seeding RAW 264.7 macrophages in 24-well tissue culture plates, cells were infected with BCG at an MOI of 10 for 2 h at 37 °C in a humidified atmosphere containing 5% CO_2_. Following infection, extracellular bacteria were eliminated by treatment with amikacin (20 μg/mL, Sigma-Aldrich, St. Louis, MO, USA) for 45 min. Cells were then washed twice with sterile phosphate-buffered saline (PBS) to remove residual extracellular bacteria. Fresh complete culture medium containing the indicated compounds was subsequently added, while untreated infected cells received fresh medium alone.

For the Day 0 (d0) time point, cells were harvested immediately after the washing step. Twenty- four hours later, cells were washed once with PBS and lysed with 0.1% Triton X-100 in PBS to release intracellular bacteria. Cell lysates were serially diluted 10-fold in sterile PBS, and appropriate dilutions were plated in duplicate onto Middlebrook 7H10 agar supplemented with 10% OADC enrichment. The plates were incubated at 37 °C for 3–4 weeks, after which visible colonies were counted manually. The intracellular bacterial burden was expressed as colony-forming units per milliliter (CFU/mL).

### Small interfering RNA transfection

RNA interference was used to investigate the role of FOXO3 in regulating IL-6 expression. TDMs were seeded at 2×10^5^ cells/well in 24 well plates and transfected with 50 nM of either FOXO3-spesefic siRNA, or a non-targeting control siRNA (siCT) (siGENOME SMARTpool, Thermo Fisher Scientific, Lafayette, CO, USA), using Lipofectamine RNAi MAX reagent (Thermo Fisher Scientific, Waltham, MA, USA), following the manufacturer’s optimized protocol. Forty-eight hours after transfection, cells were infected with BCG (MOI 10) for 24h. Gene silencing efficiency was assessed at both the transcriptional level by qRT-PCR and at the protein level by western blot analysis.

### Real time quantitative PCR

Total RNA was extracted from treated cells using TRIzol^®^ reagent (Invitrogen) and further purified with the RNeasy Mini Kit (Qiagen, Hilden, Germany) according to the manufacturer’s protocol. Complementary DNA (cDNA) was synthesized using M-MLV reverse transcriptase enzyme (Invitrogen, Carlsbad, CA, USA). Real-time quantitative PCR was performed using the platinum^®^ Syber Green qPCR Supermix-UDGw/ROX (Life Technologies, Carlsbad, CA, USA). Gene expression levels were normalized to β-actin and GAPDH, and relative changes were calculated using the target threshold cycle value (Ct) and the 2−ΔΔCt method. Primer sequences used for qPCR are provided in the supplemental text (**Supplementary Table 1**).

### Gel electrophoresis and immunoblotting

To assess the expression levels and the phosphorylation statue of AKT and FOXO3, TDMs were maintained as described above. Total proteins were isolated using M-PER™ Mammalian Protein Extraction Reagent (Thermo Fisher Scientific, Rockford, IL, USA), and protein concentration was determined using Bicinchoninic Acid Protein Assay (BCA, Sigma-Aldrich, St. Louis, MS, USA). Twenty micrograms of total cell protein were resolved by 4-10% SDS-PAGE, transferred to a PVDF membrane and probed with antibodies directed against AKT (1:1000, Cell Signaling Technology, Danvers, MA, USA #9272), p-Akt ^Ser473^ (1:1000, Cell Signaling Technology, Danvers, MA, USA #4060), FKHRL-1 (FOXO3, 1:1000) and FKHRL1-D12 (p-FOXO3^Thr32^,1:1000). β-actin (1:2000, Sigma Aldrich, Merck, St. Louis, MO, USA) was used as a loading control. The protein bands were detected by ECL™ Cytvia (Amersham, Little Chalfont, Buckinghamshire, UK) and visualized by FUSION Solo s chemiluminescence imaging system (Vilber, France). Densiometric quantification was performed using ImageJ bundled with 64-bit Java 8.

### Enzyme linked immunosorbent assay

The concentration of IL-6 in cell culture supernatants was quantified using a commercial ELISA kit (BD Biosciences, San Jose, CA, USA) according to the manufacturer’s instructions. Briefly, samples and standards were incubated in wells pre-coated with anti-IL-6 capture antibody, followed by detection with anti-IL-6 biotinylated antibody and streptavidin H-RP. The enzymatic reaction was developed with 3,3′,5,5′-Tetramethylbenzidine (TMB) substrate and stopped with sulfuric acid. Absorbance was measured at 450 nm with a reference correction at 570 nm using a microplate reader. Cytokine concentrations were calculated using a standard curve generated with recombinant IL-6.

### *In silico* analysis and plasmids construction

Putative FOXO3 binding sites within the human IL-6 promoter were identified using the MatInspector analysis tool of the Genomatix software (https://www.eurenomics.eu/the-group/consortium/genomatix/index.html) and cross validated with the Eukaryotic Promoter Database (EPD). Four promoter fragments, corresponding to the -600 bp, -900 bp, 1.5 kb, and 2 kb upstream of the transcription start site, were PCR amplified from human genomic DNA using specific primers (listed in **Supplementary Table 2**) incorporating restriction enzyme sites. The amplified products were gel-purified, digested with appropriate restriction enzymes and ligated into the pGL-3 basic luciferase reporter vector. Positive clones were screened by colony PCR and restriction enzyme digestion. Verified plasmids were prepared in endotoxin-free form for luciferase reporter assays.

### Gene reporter assay/Luciferase assays

Transient transfections of HEK 293 cells were performed as previously described (21), using Lipofectamine 2000 (Thermo Fisher Scientific, Waltham, MA, USA). Briefly, to assess IL-6 promoter activity, 0.8 µg of IL-6 promoter-pGL3 basic reporter constructs containing various lengths of the IL-6 promoter region (-600 bp, -900 bp, -1.5 kb, or -2 kb) were transfected per condition. For co-transfection experiments, cells were additionally received 0.5 µg of either the pEGFP control vector encoding for the green fluorescent protein (GFP) or the pEGFP-FOXO3TM encoding for GFP-FOXO3 triple mutant (GFP-FOXO3TM, a constitutive active form of FOXO3 where the three Akt phosphorylation sites were mutated). Six hours post-transfection, cells were either mock-treated or infected with BCG at an MOI of 1:10 in order to assure the infection of all cells (21). For transfection experiments with the -600 bp and -900 bp IL-6 promoter constructs, cells were treated 6h-post transfection with Wortmanin (100 nM) (Sigma-Aldrich, St. Louis, MO, USA) or MK-2206 (5 µM) to inhibit the PI3K/AKT pathway and thereby promote FOXO3 activation. Neither the used MOI nor the inhibitors presented any toxicity or effect on cell viability as previously reported (21, 23). Cultures maintained in medium supplemented with 20% FBS served to activate the PI3K/AKT pathway, resulting in FOXO3 inhibition. After 24h, cells were lysed in Passive Lysis Buffer (Promega, Madison, WI, USA), and luciferase activity was measured using the Dual-Luciferase Reporter Assay System (Promega, Madison, WI, USA) on a Thermoscientific Varioscan Flask Plate (Promega, Madison, WI, USA). Luciferase activity was normalized to the protein concentration of the total cell lysate, determined by BCA assay, and further adjusted relative to GFP expression levels to control for variations in transfection efficiency. Promoter activities were shown as Relative Light Unit (RLU).

### Site directed mutagenesis of the IL-6 promoter

Site directed mutagenesis was performed on the -900bp, -1.5kb, and -2kb IL-6 promoter constructs to specifically alter the predicted FOXO3 binding sites identified by in *silico* analysis (relative to Genomatix and EPD). Mutagenesis was carried out as previously reported in (21), using the Quick-change XL site directed mutagenesis kit (Agilent technology) according to the manufacturer’s instructions. Briefly, mutagenic primers were designed to disrupt FOXO3 binding motifs within the IL-6 promoter sequence contained in the reporter plasmid (primers listed in **Supplementary Table 3**). The three predicted FOXO3 binding sites were mutated as follows: Mutant 1, Mutant 2 and Mutant 3 were constructed by deleting TTA (-786 position), AAC (-1331 position) and AAT (-1631 position), respectively. PCR amplification was followed by DpnI digestion, and the resulting mutated plasmids were transformed into XL-1 blue *Escherichia coli* competent cells. Positive clones were initially screened by restriction enzyme digestion and subsequently confirmed by Sanger sequencing. The impact of each mutant on IL-6 promoter transcriptional activity was evaluated using gene reporter assay described above.

### Statistical analysis

Statistical analysis was performed using GraphPad Prism version 8.4.3 (GraphPad Software, San Diego, CA, USA). Simple comparisons between two groups were analyzed using an unpaired Student’s *t*-test, whereas multiple comparisons were performed using an analysis for variance (ANOVA). Significance is indicated by a symbol. Differences with a *p*-value < 0.05 were considered statistically significant.

## Results

### PI3K/Akt/FOXO3 axis regulates IL-6 expression in BCG-infected macrophages

**Figure 1:**
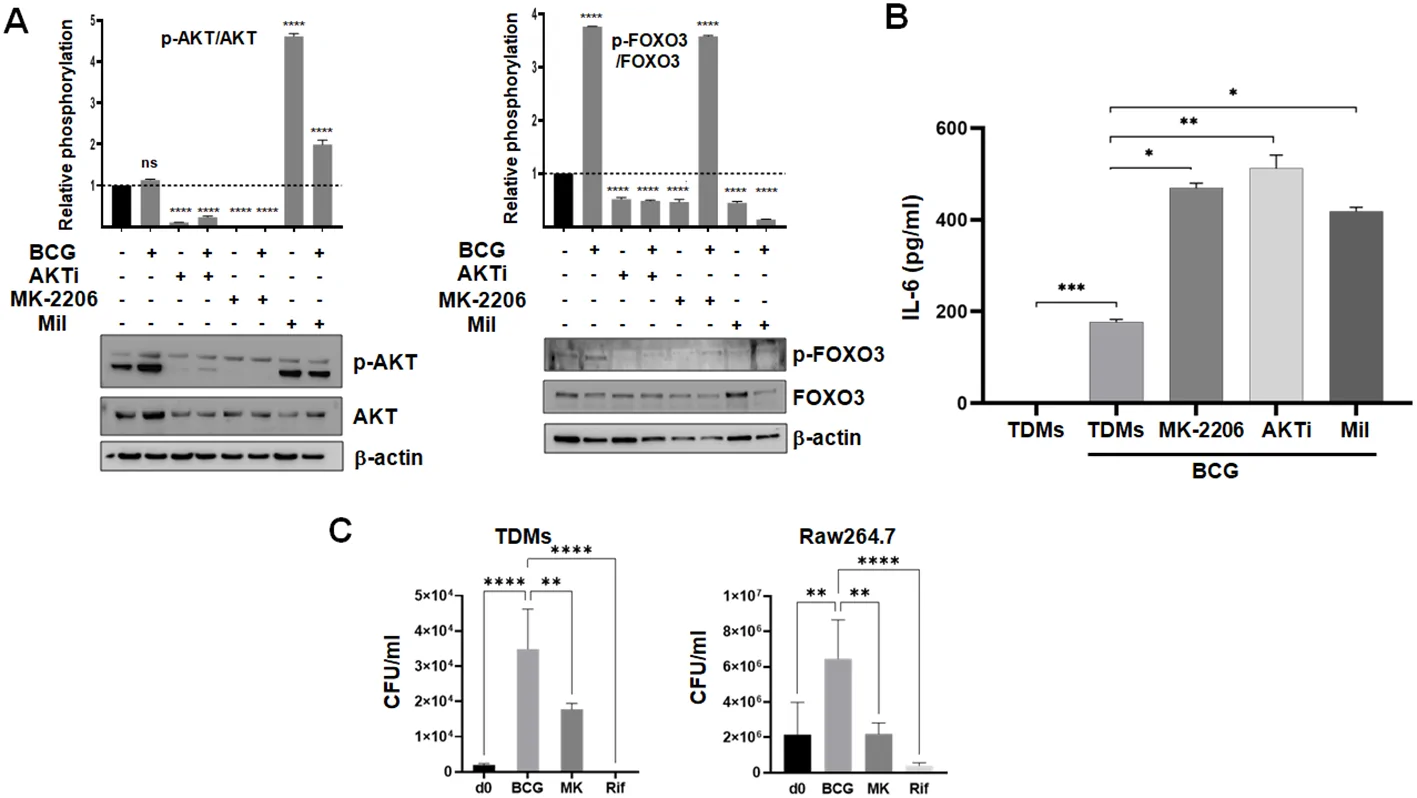
The PI3K/Akt axis regulates BCG-induced IL-6 secretion in macrophages. TDMs were infected with BCG for 2 hours at an MOI of 10. Cells were than washed and treated with the PI3K/Akt inhibitors MK-2206 (5 µM), AKTi (5 µM) or Miltefosine (3 µM) for 24h. **(A)** Inactivation of PI3K/Akt signaling pathway was analyzed by Western blot. Cell lysates were probed with antibodies against phosphorylated Akt (Ser473 p-Akt), total Akt, phosphorylated FOXO3 (T32 p-FOXO3) and total FOXO3. β-actin was used as a loading control. Representative blots from two independent experiments are shown. **(B)** IL-6 secretion in culture supernatant was quantified by ELISA following BCG infection for 24h in the presence or absence of PI3K/Akt inhibitors. Data are expressed as mean ± SEM from three independent experiments. **(C)** Effect of AKT inhibition/FOXO3 activation, using MK-2206 (MK), on bacterial survival assessed in two macrophage cell lines, the human TDMs and murine RAW264.7. Rifampicine (Rif) was used as positive control. Data are expressed as mean ± SEM from three independent experiments. Statistical significance was determined using one way ANOVA test. A p value <0.05 was considered statistically significant. (*p < 0.05; **p < 0.01; ***p < 0.001; ****p < 0.0001).

**Figure 2:**
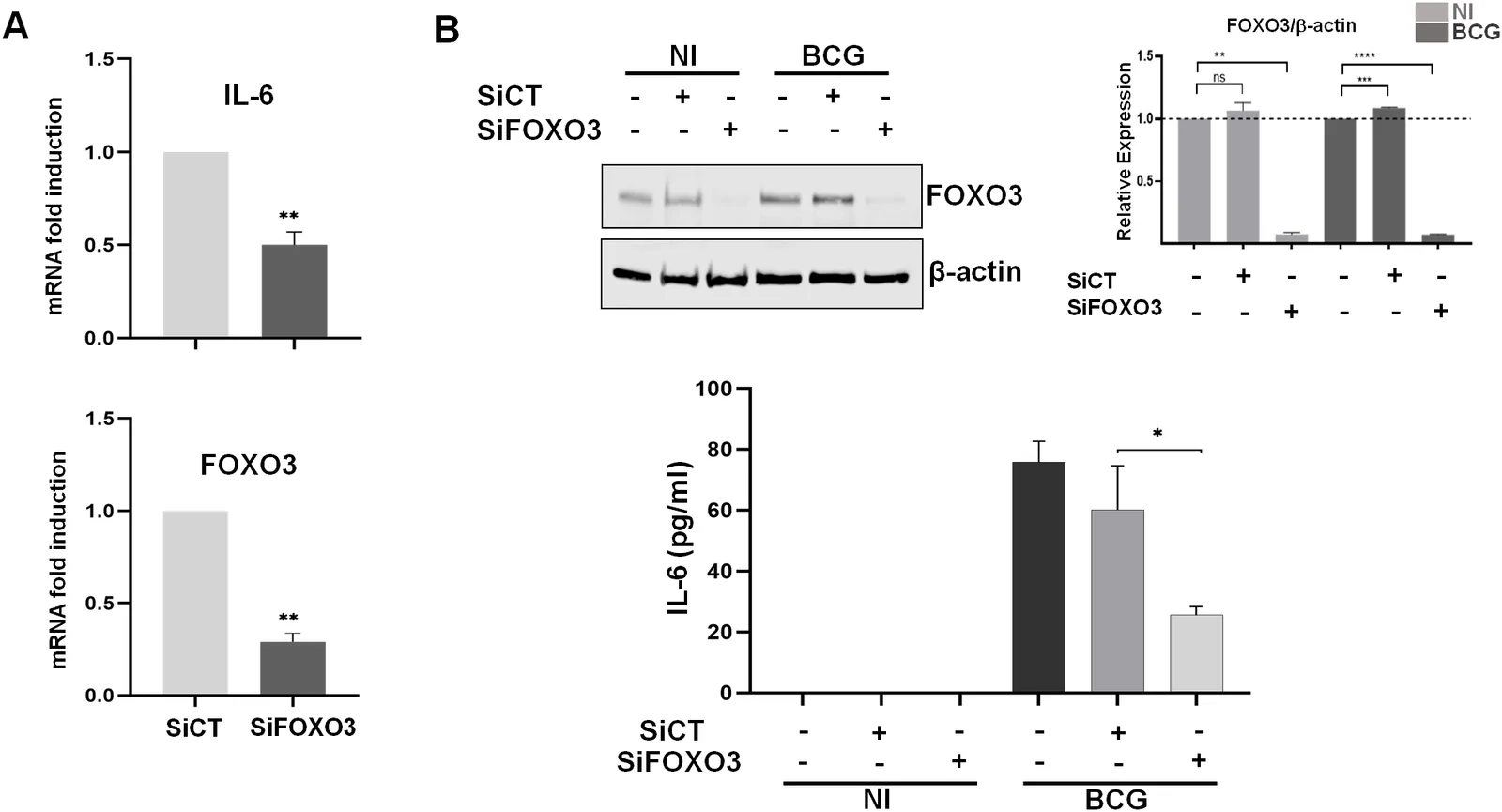
Knockdown of FOXO3 reduces IL-6 expression in BCG- infected macrophages. TDMs were transfected with FOXO3 specific siRNA (siFOXO3) or scrambled control siRNA (siCT) for 48h and subsequently infected with BCG for 24h. **(A)** Relative mRNA expression levels of FOXO3 and IL-6 were quantified by Real Time Quantitative PCR (RT-PCR) and expressed as fold induction after normalization. **(B)** IL-6 secretion, in culture supernatants, quantified by ELISA (lower panel). FOXO3 knockdown efficiency was verified by western blot analysis using antibodies against FOXO3, with the β-actin as a loading control (upper panel). Data are presented as mean ± SEM from at least three independent experiments (*p<0.05; **p<0.01; ***p < 0.001, ****p < 0.0001).

In macrophages, IL-6 production has been shown to depend on PI3K/Akt activation (20). Because FOXO3 is a major downstream target that is directly phosphorylated and inhibited by Akt, we sought to determine whether pharmacological inhibition of the PI3K/Akt pathway affects FOXO3 activation and the BCG-induced secretion of IL-6. To address this question, we used three pharmacological inhibitors targeting different levels of the pathway. MK-2206 (MK), a highly specific non-ATP-competitive allosteric inhibitor of Akt activation and Akt inhibitor (Akti), which directly blocks Akt activity through inhibition of its catalytic domain. In addition, we used the Miltefosine (Mil), an alkyl phosphocholine compound that inhibits the PI3K/Akt axis upstream by disrupting membrane lipid organization and PI3K recruitment. As expected, treatment with MK and Akti caused a marked reduction in Akt phosphorylation, whereas Mil did not affect Akt phosphorylation (**Figure 1A**). We also observed that pretreatments with MK, Akti, and Mil induced FOXO3 dephosphorylation, suggesting its activation (**Figure 1A**). Consistent with FOXO3 activation, ELISA quantification showed that IL-6 secretion was markedly increased in BCG infected macrophages treated with MK, Akti, or Mil (**Figure 1B**). We have previously showed that FOXO3 activation in macrophages induced a pro-inflammatory M1 phenotype, enhancing the bactericidal effect of the cells (21). We have then assessed the mycobacteria survival inside the MK-treated TDMs and Raw264.7 macrophages. Treatment with AKT inhibitor MK led to an enhancement of the killing of mycobacteria by the both treated cells (**Figure 1C**). Together, these findings demonstrate that PI3K/Akt signaling negatively regulates IL-6 expression during BCG infection, likely via inhibition of FOXO3, and that pharmacological blockade of this pathway activates FOXO3, leading to IL-6 induction. To investigate whether FOXO3 directly regulates IL-6 expression, TDMs were transfected with a pool of small interfering RNA (siRNA) to silence FOXO3 expression. The efficiency of FOXO3 knockdown was confirmed at both the mRNA and protein levels by qRT-PCR and immunoblotting, respectively (**Figure 2**). Silencing of FOXO3 in TDMs significantly reduced BCG-induced IL-6 mRNA expression compared to scrambled siRNA (SiCT) controls, indicating that FOXO3 acts as a transcriptional activator of IL-6 (**Figure 2A**). This decrease at the transcription level was accompanied by a corresponding reduction in IL-6 protein secretion (**Figure 2B**). Overall, these findings demonstrate that FOXO3 significantly contributes to IL-6 expression in BCG-infected macrophages by activating its transcription.

### FOXO3 directly activates IL-6 promoter activity in BCG infected cells

**Figure 3:**
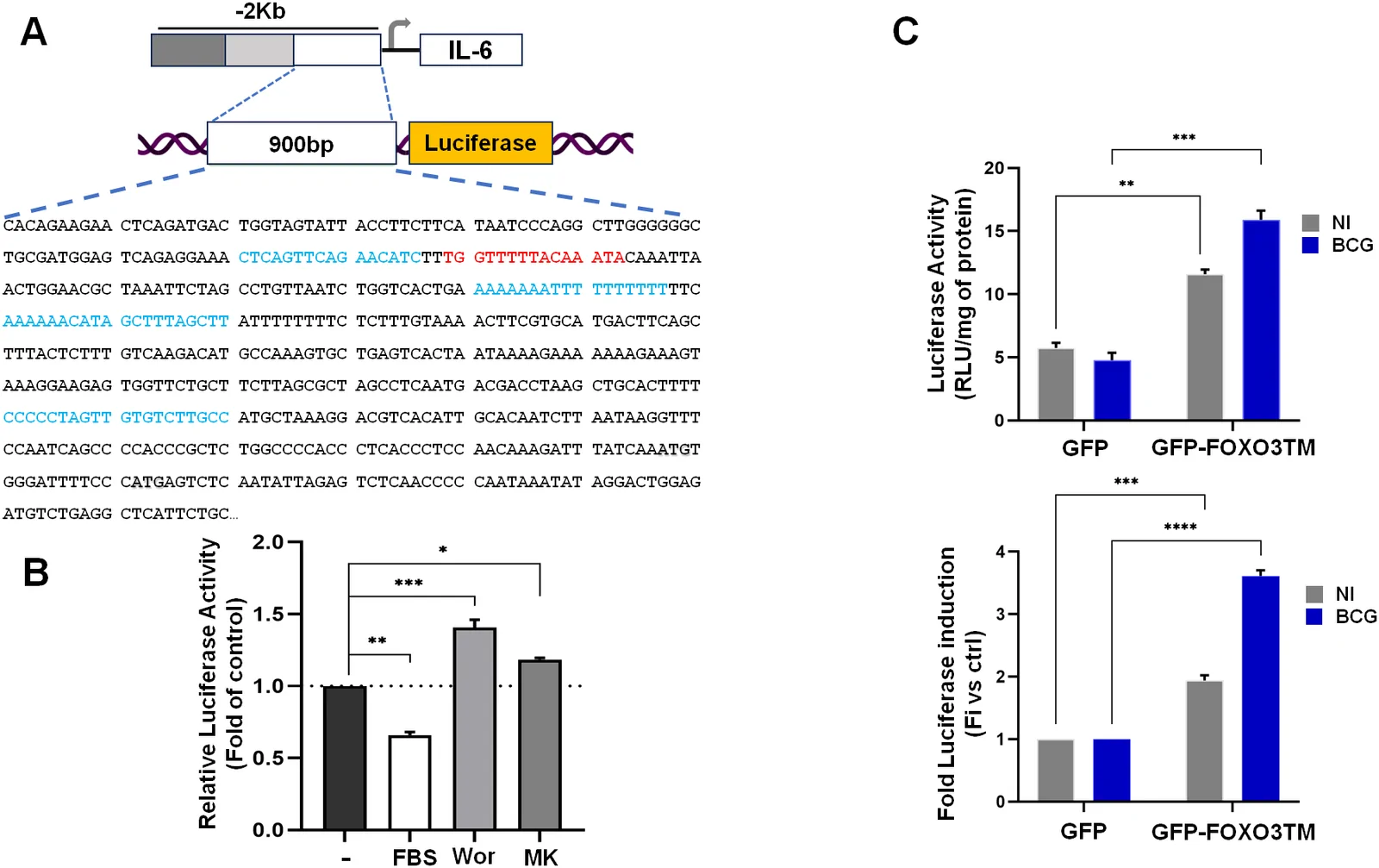
FOXO3 directly activates the -900 bp IL-6 promoter activity in BCG infected cells. **(A)** Schematic representation of the -900 bp region of the human IL-6 promoter showing the location of five putative Forkhead DNA binding elements identified using the Genomatix algorithm. The sequence highlighted in red corresponds to FOXO3 binding motif identified by the Eucaryotic Promoter Database (EPD). **(B)** Luciferase reporter assays were performed in HEK 293 cells transfected with the proximal -900 bp IL-6 promoter construct. Six hours post-transfection, cells were treated with the PI3K inhibitor Wortmanin (Wort) or the Akt inhibitor MK-2206 (MK) and thereby promote the activation of the endogenous FOXO3. Parallel cultures were transfected with the same construct and maintained in medium containing 20% FBS to sustain PI3K/Akt activation and consequently inhibit FOXO3 function. Luciferase activity was measured and expressed as fold induction relative to control conditions (i.e., non-treated cells). **(C)** HEK 293 cells were co-transfected with GFP control or GFP-FOXO3TM together with the pGL3-IL6 promoter (-900 bp) reporter construct. Luciferase activity was quantified and expressed as fold induction relative to control transfections. Luciferase activity was only measured in GFP positive cells, as indicated in materials and method section. The data correspond to the mean ± SEM of three different experiments (*p<0.05; **p<0.01; ***p<0.001; ****p<0.0001).

**Figure 4:**
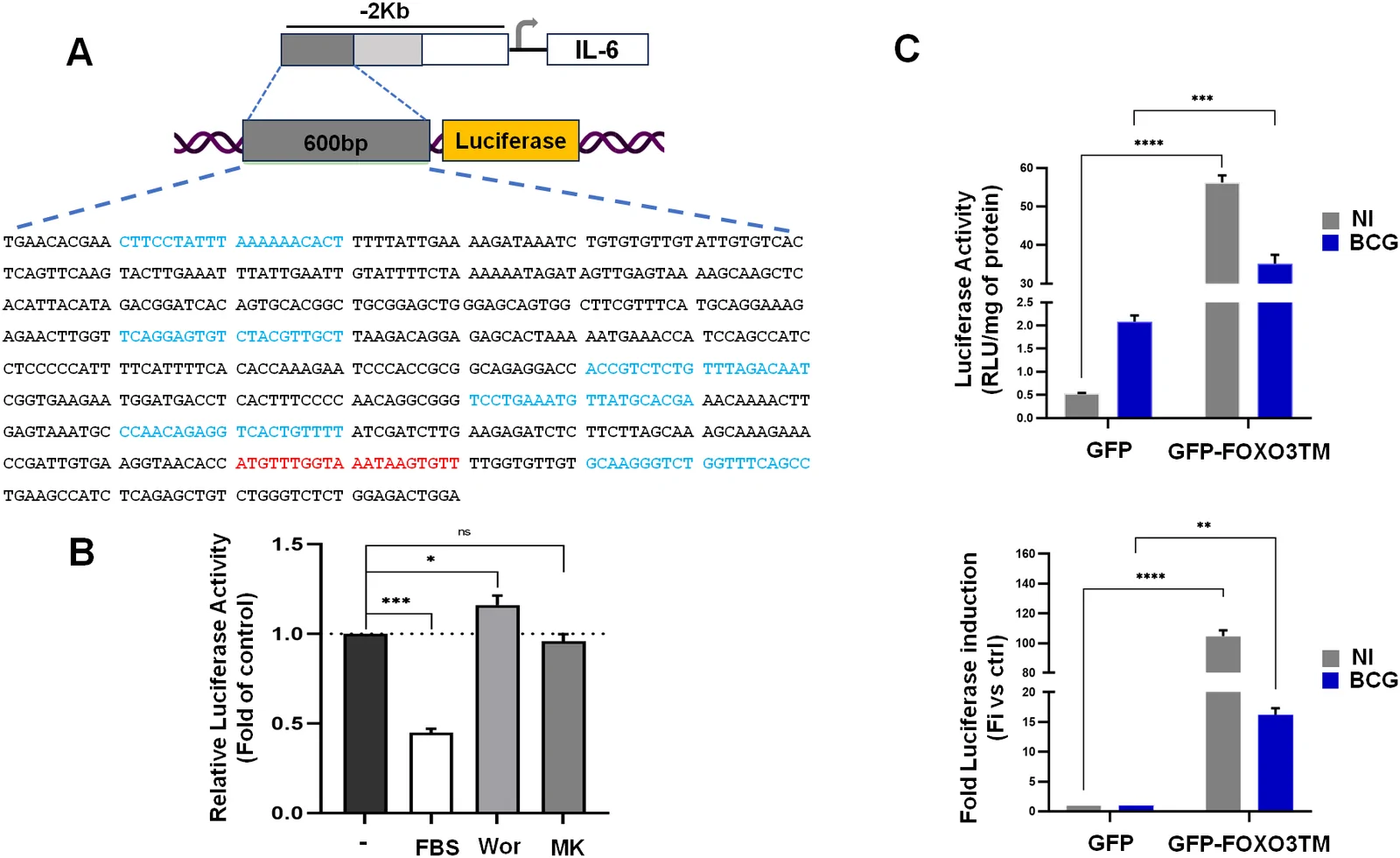
FOXO3 directly activates the distant 600 bp IL-6 promoter activity in BCG infected cells. **(A)** Schematic representation of the 600 bp region of the human IL-6 promoter showing the location of seven putative Forkhead DNA binding elements identified using the Genomatix algorithm. The sequence highlighted in red corresponds to FOXO3 binding motif identified by the Eucaryotic Promoter Database (EPD). **(B)** Luciferase reporter assays were performed in HEK 293 cells transfected with either the distal 600 bp IL-6 promoter construct. Six hours post-transfection, cells were treated with the PI3K inhibitor Wortmanin (Wort) or the Akt inhibitor MK and thereby promote the activation of the endogenous FOXO3. Parallel cultures were transfected with both constructs and maintained in medium containing 20% FBS to sustain PI3K/Akt activation and consequently suppress FOXO3 function. Luciferase activity was measured and expressed as fold induction relative to control conditions. **(C)** HEK 293 cells were co-transfected with GFP control or GFP-FOXO3TM together with the pGL3-IL6 promoter (600bp) reporter construct. Luciferase activity was quantified and expressed as fold induction relative to control transfections. The data correspond to the mean ± SEM of three different experiments (*p<0.05; **p<0.01; ***p<0.001; ****p<0.0001).

To further characterize the FOXO3-mediated regulation of IL-6 transcription, we conducted an *in-silico* analysis of the human IL-6 promoter using the MatInspector tool (Genomatix) in combination with the Eukaryotic Promoter Database (EPD). The Genomatix platform provided two annotated versions of the IL-6 promoter: a longer proximal promoter fragment of approximately 900 bp upstream of the transcription start site (TSS) (**Figure 3A**), and a distant shorter version of about 600 bp (**Figure 4A**). Analysis of the 900bp sequence revealed five putative Forkhead DNA-binding elements located at positions -530, -710, -730, -786 and -810. Notably, the motif at -786 corresponds to a typical FOXO3 binding site, as independently confirmed using the EPD (**Figure 3A**). Analysis of the -600 bp IL-6 promoter fragment revealed seven putative Forkhead binding motifs at positions -1613, -1631, -1723, -1763, -1823, -1933 and -2143. Importantly, the element at -1631 was predicted to be a typical FOXO3 binding site, consistent with EPD (**Figure 4A**). Together, these *in silico* analysis indicate that IL-6 promoter contains multiple Forkhead-DNA elements across both annotated promoter versions, including at least one typical conserved FOXO3-binding site, supporting the hypothesis that IL-6 may be a direct transcriptional target of FOXO3.

To evaluate the contribution of FOXO3 to IL-6 transcriptional regulation across different promoter lengths, we performed a gene reporter assay using HEK293 cells transfected with either the proximal -900 bp or the distant -600 bp IL-6 promoter constructs. Six hours post-transfection with each promoter construct, cells were treated with the PI3K inhibitor Wortmanin (Wort) or the Akt inhibitor MK and thereby promoted the activation of the endogenous FOXO3. Parallel cultures were transfected with both constructs and maintained in medium containing 20% FBS to sustain PI3K/Akt activation and consequently suppress FOXO3 function. Luciferase activity was measured 24 hours after the indicated treatments to assess the IL-6 promoter activity. We first examined the -900 bp IL-6 promoter fragment. Inhibition of the PI3K/Akt pathway with either Wort or MK led to a robust increase in reporter activity, indicating that FOXO3 activation enhances transcription driven by this extended promoter region (**Figure 3B**). In contrast, high-serum conditions markedly reduced promoter activity, consistent with FOXO3 inactivation. We next evaluated the distant -600 bp IL-6 promoter construct. Wort treatment significantly increased reporter activity, whereas Mk did not produce a statistical effect. Consistent with these findings, 20% FBS suppressed promoter activity (**Figure 4B**). We next performed a gene reporter assay in HEK293 cells, as detailed in the methods. In this experiment, cells were co-transfected with either the pEGFP control vector or with pEGFP-FOXO3TM plasmid encoding for the constitutively active form of FOXO3 along with the -900 bp or -600 bp IL-6 luciferase construct. For the proximal -900 bp IL-6 promoter construct, overexpression of FOXO3 elicited a ~4-fold increase in luciferase activity in BCG-infected HEK293 cells compared with GFP control, indicating that FOXO3 markedly enhances promoter activation during infection (**Figure 3C**). For the distant -600 bp promoter construct, overexpression of FOXO3 significantly amplified the BCG-induced promoter activity, resulting in an approximately 15-fold increase in luciferase signal compared with GFP, confirming that FOXO3 strongly potentiates IL-6 transcription within this shorter promoter fragment (**Figure 4C**).

### FOXO3 exerts dual regulation effects on IL-6 transcription responsive elements

**Figure 5:**
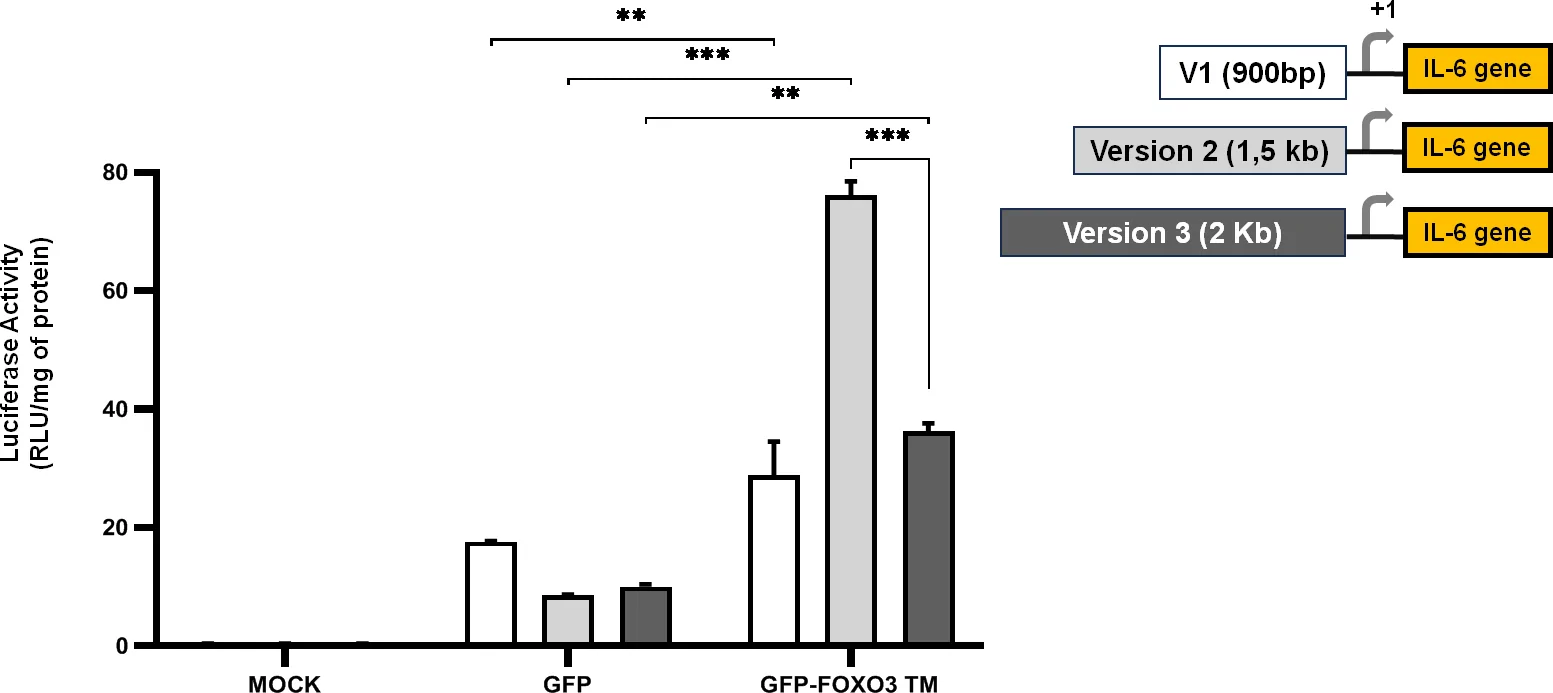
FOXO3 differentially regulates IL-6 promoter activity depending on promoter length. HEK 293 Cells were co-transfected with luciferase reporter constructs containing 900 bp, 1.5 kb or 2 kb regions of the human IL-6 promoter together with either GFP control or constrictively active FOXO3TM expression vectors. Promoter activity is expressed as relative luciferase units (RLU). Data are presented as mean ± SEM of three different experiments (**p<0.01; ***p<0.001).

Since the distant promoter construct exhibited an unusually strong response than the proximal fragment (**Figure 4C**), we aimed to examine the contribution of each individual promoter segment in a more physiological context. To this end, we generated two additional IL-6 promoter constructs, V2 (1.5 kb) and V3 (2 kb) and assessed their role in FOXO3-mediated regulation of IL-6 transcription (**Figure 5**).

As expected, overexpression of FOXO3 moderately increased luciferase activity under the control of the V1 fragment, indicating that the proximal promoter contains functional FOXO3 responsive elements but provides only limited transcriptional activation (**Figure 5**). Extending this sequence to 1.5kb produced the strongest luciferase activity among all constructs, pointing to the presence of upstream regulatory elements that markedly amplify FOXO3-driven transcription. The 2 kb (V3) construct also responded to the active FOXO3, but its activity was lower than the one of V2. This indicates a negative regulation exerted by the additional 0.5 kb segment rather than an enhancer effect as previously reported (25). Together, these data demonstrate that FOXO3 directly enhances IL-6 promoter activity and identify the 1.5 kb region as the most potent regulatory region for FOXO3 mediated transcription, while the distant 0.5 kb fragment (V3) modulate such activity.

**Figure 6:**
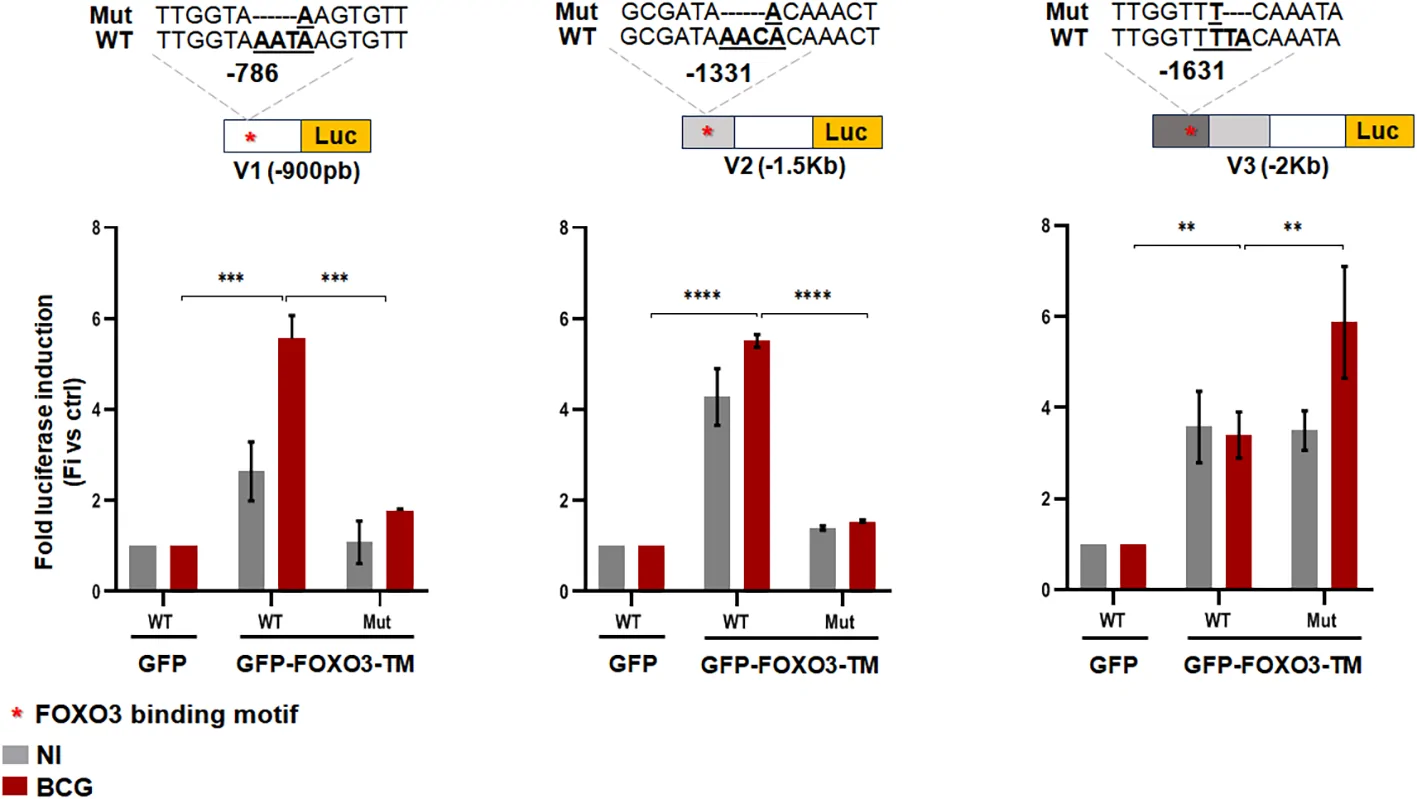
Functional contribution of individual forkhead DNA binding elements to FOXO3 mediated IL-6 promoter activity. To assess the functional contribution of individual Forkhead DNA binding elements, site directed mutagenesis were performed on the IL-6 promoter to generate three mutant constructs at positions -786, -1331 and -1631. Schematic representation and gene reporter assay performed using the wild type (WT) IL-6 promoter and three mutant constructs. Six hours after transfection, cells were infected or not with BCG, and luciferase activity was measured 24h later. Luciferase activity was quantified and expressed as fold induction relative to control transfections. Data are presented ± SEM of three different experiments (**p<0.01; ***p<0.001; ****p<0.0001).

To further investigate the functional contribution of FKHR/FOXO3 binding elements and identify the role of each motif to the FOXO3-mediated regulation of the IL-6 promoter, we generated three IL-6 promoter mutants targeting the putative FOXO3 binding motifs at positions -786, -1331, and -1631 (**Figure 6**). Luciferase assay revealed that disruption of the FOXO3 motif at -786 site of the proximal V1 portion led to a significant reduction in FOXO3-dependent activation, indicating that this element is the direct target through which FOXO3 activates the transcription of IL-6 gene. Similarly, disruption of FOXO3 motif at -1331 site of the V2 fragment almost completely abolished FOXO3-mediated induction of IL-6 transcription. However, mutation of FOXO3 motif at the -1631 site of the distant 600 bp fragment did not affect the FOXO3-induced activity in non-infected cells, but rather enhanced it in BCG-infected ones. This indicates that this distant FOXO3 site exerts a negative regulatory effect on IL-6 transcription. Together, these results indicate that FOXO3-dependent activation of the IL-6 promoter pass predominantly through the binding to -786 and -1331 sites, while FOXO3 displays a negative effect on IL-6 transcription by the interaction with its motif at the -1631 position.

## Discussion

IL-6 has been shown to play a crucial role in early host defense mechanisms against TB and also shapes the adaptive immune response. Uncovering the cellular effectors regulating its expression would help developing host-directed tools to either modulate its detrimental effects or boost its beneficial ones, especially in the context of TB treatment and vaccination.

In the present study, we identified FOXO3 as a critical and bifunctional regulator of IL-6 transcription, in the context of BCG infection.

We first assessed, in BCG-infected macrophages, the involvement of the PI3K/AKT pathway, a negative regulator of FOXO3 activity, in IL-6 expression. We found that inhibition of Akt led to FOXO3 dephosphorylation along with an enhancement of IL-6 secretion, suggesting a positive regulation exerted by FOXO3 on IL-6 expression. These data are in agreement with the previous report indicating a regulation of IL-6 expression by the canonical PI3K/Akt pathway (19, 20). FOXO3 activation in macrophages also led to the enhancement of the bactericidal effect of the infected cells. This data correlates with our previous study where we found that FOXO3 activation directed the differentiation of macrophages toward a pro-inflammatory M1 phenotype with a higher killing effect against the infecting mycobacteria (21). This suggests that FOXO3-mediated activation of IL-6 may contribute to the generation of M1 phenotype of macrophages, enhancing their bactericidal effect. To further check if IL-6 expression passes through a direct effect of FOXO3, we silenced its expression in macrophages. Absence of FOXO3 led to a significant decrease of IL-6 transcription and secretion in BCG-infected macrophages, revealing a direct regulation of IL-6 production by FOXO3. This data correlates with our previous study where the knocking down of FOXO3 in primary macrophages led to a Th2 immune response characterized by an increase in IL-10 production and a decrease in IL-6 (21). Moreover, this new described positive regulation of IL-6 by FOXO3 may have contributed to the establishment of a robust adaptive immune response seen in mice treated with FOXO3 activator during BCG vaccination (22). Our data is also supported by a previous report showing the protective effect, exerted by FOXO3, against *Salmonella typhimirium* infection through the induction of an IL-6-mediated adaptive Th1 response (26).

To further characterize the mechanisms of FOXO3-mediated transcriptional regulation of IL-6, we performed gene regulation assays, using different regions of IL-6 promoters, along with mutagenesis experiments performed on FOXO3 motifs localized in each promoter region. Genomatix software proposed two promoter regions of IL-6. Interestingly, we found that FOXO3 significantly induces the luciferase activity of both the proximal 900 bp fragment and the distant 600bp one, with much stronger induction at this latter fragment This may be explained by the fact that the proximal 900 bp fragment contains all the functional cis-regulatory elements required for its finely transcriptional activation including a nuclear factor kappa B sites (NF-kB), two C/EBP B sites (also known as NF-IL-6), two SP1 sites, a cyclic AMP response element binding protein (CREB) site, an interferon regulatory factor (IRF) site, and an activator protein-1 (AP-1). Among these, the AP-1 site bound by C-Jun represents the most critical regulatory element, acting in cooperation with NF-kB to drive IL-6 transcription (27). In contrast, the shorter 600bp distal promoter version appears to lack several of these essential regulatory elements. Consequently, the 900bp promoter version seems to represent a more physiologic region.

In order to further investigate the transcriptional regulation, mediated by FOXO3 on IL-6, in a more physiological context, we have generated two additional constructs to delineate the effect of each promoter region along with the contribution of each FOXO3 motif, as assessed by mutagenesis experiments. We found that both proximal 900 bp (V1 construct) and 1.5 kb (V2 construct) fragments mediated the FOXO3-positive regulation of IL-6 with a higher effect with the longer V2 fragment construct. This suggests an additional effect of FOXO3 motifs since the longer fragment contains two motifs located at -786 and -1331 positions. Disruption of FOXO3 motif located at -786 of V1 construct led to total abrogation of FOXO3 effect on IL-6 transcription. Surprisingly, we found the same effect when the FOXO3 motif at -1331 position of the V2 construct -containing also the V1 fragment- was invalidated. Indeed, we did not expect the total abolition of FOXO3 effect by the sole disruption of its motif at -1331 position, since we did not modify the other one at position -786. This data indicates that the additional transcriptional effect of the V2 construct relies on the presence of the two motifs at -1331 and -786 positions that would spatially coordinate and interact with FOXO3 to induce an optimal induction of IL-6 transcription. The second explanation would be that the extension of the fragment with an addition 600 bp portion would cause spatial changes of the chromatin leading to the hiding of FOXO3 motif at position -786. This could represent one way to finely control the IL-6 expression by either exposing or hiding FOXO3 motifs located in different regions of the IL-6 promoter.

To further characterize the regulation of IL-6 transcription, we extended the V2 construct (1.5 kb fragment upstream of the TSS) with the 600 bp distant portion. This extension, which included a proximal fragment containing seven forkhead responsive elements, did not enhance luciferase expression, instead, it decreased it. This data suggests that this portion of the promoter exerts an inhibitory effect mediated by FOXO3 on IL-6 transcription. This was further supported by the increase of the luciferase activity when FOXO3 motif at -1631 position was disrupted. This negative regulation of IL-6 expression by FOXO3 is in agreement with a previous report showing that activated T cells from FOXO3 KO mice secrete higher amount of IL-6 than the wild type (28), even though the cell types are not the same.

**Figure 7:**
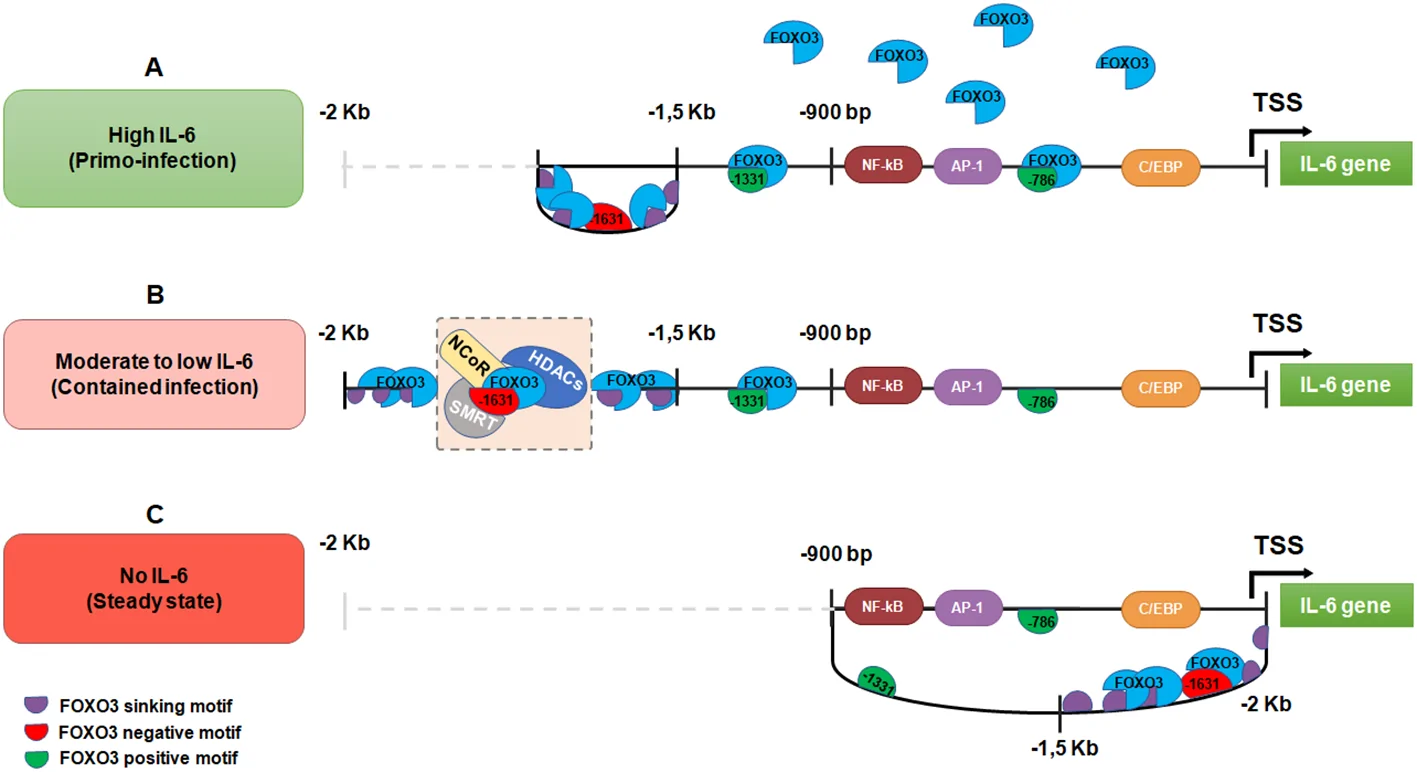
FOXO3 finely tunes IL-6 expression to boost the adaptive immune response against *Mtb* while preventing excessive inflammation. **(A)** In the context of primo-infection, FOXO3 binds to its positive IL-6-inducers motifs at the 1.5kb fragment (-786 and -1331 motifs), while the chromatin remodeling blocks its access to the negative motif at -1631 position, leading to a high expression of IL-6. **(B)** In order to overcome an excessive detrimental inflammatory response, FOXO3 would dissociate from those positive regulating motifs, leaving the control of IL-6 transcription at the proximal region to the cis-regulatory elements such as the C/EBP B, SP1 CREB, IRF1 and AP1. Simultaneously, FOXO3 will bind to its negative IL-6 regulator motif -1631 at the distant 0.5kb fragment, which may also contain specific sequence motifs or chromatin features that favor the recruitment of co-repressors, such as Histone Deacytelases (HDACs) or the Nuclear Receptor Co-Repressor/Silencing Mediator for Retinol and Thyroid Hormone Receptors (NCoR/SMRT) complex, over co-activators. This scenario would then lead to a moderate or a weak expression of IL-6. The binding of FOXO3 at the distal 0.5kb site might facilitate the formation of a DNA loop that physically interferes with the proximal activation complex. Such “loop-mediated repression” could sequester the proximal promoter or the TSS away from the active transcriptional machinery. Finally, the distal 0.5kb fragment, containing seven forkhead binding motifs, may also act as a sink for FOXO3 or its associated activators, effectively reducing their local concentration at the proximal promoter and thereby dampening the overall transcriptional output. **(C)** A final configuration of the promoter would lead to a total blockade of IL-6 transcription through both the negative above indicated regulation mechanisms and by a major remodeling of the chromatin leading to a locked configuration of IL-6 promoter.

The ability of a transcription factor to exert opposing effects on the same gene is a sophisticated regulatory strategy, particularly crucial in immune responses where precise cytokine regulation is paramount. We propose several non-mutually exclusive mechanisms to explain this phenomenon in the context of IL-6 during mycobacterium infection:

In the context of primoinfection, an IL-6-mediated pro-inflammatory response would be needed to control infection and orchestrate the adaptive immune response. Signaling pathways, activated by pathogen recognition receptors, could influence the availability or activity of FOXO3 co-factors to favor a high expression of IL-6. FOXO3 will therefore bind to its positive IL-6-inducers motifs at the 1.5kb fragment (i e, -786 and -1331 motifs), while the chromatin remodeling blocks the access to the FOXO3 negative motif at -1631 position (**Figure 7**). In order to overcome an excessive detrimental inflammatory response, FOXO3 would dissociate from those positive regulating motifs, leaving the control of IL-6 transcription at the proximal region to the cis-regulatory elements such as the C/EBP B, SP1 CREB, IRF1 and AP1. Simultaneously, FOXO3 will bind to its negative IL-6 regulator motif -1631 at the distant 0.5 kb fragment, which may also contain specific sequence motifs or chromatin features that favor the recruitment of co-repressors, such as Histone Deacytelases (HDACs) or the Nuclear Receptor Co-Repressor/Silencing Mediator for Retinol and Thyroid Hormone Receptors (NCoR/SMRT) complex, over co-activators. This scenario would lead either to moderate or weak expression of IL-6 (**Figure 7**). It is also well-established that the functional activity of FOXO3 is modulated by its post-translational modifications and the local protein-protein interaction environment (29, 30). FOXO3 orchestrates target gene regulation through the selective amplification of enhancer activity (31).This mediates a functional transition in FOXO3, converting it from a transcriptional activator into a repressor. Moreover, the binding of FOXO3 at the distal 0.5 kb site might facilitate the formation of a DNA loop that physically interferes with the proximal activation complex. Such “loop-mediated repression” could sequester the proximal promoter or the TSS away from the active transcriptional machinery (32). Finally, the distal 0.5kb fragment, containing seven forkhead binding motifs, may also act as a sink for FOXO3 or its associated activators, effectively reducing their local concentration at the proximal promoter and thereby dampening the overall transcriptional output (**Figure 7**). A final configuration of the promoter would lead to a total blockade of IL-6 transcription through both the negative above indicated regulation mechanisms and by a major remodeling of the chromatin leading to a locked configuration of IL-6 promoter (**Figure 7**).

The present study identifies FOXO3 as a critical and bifunctional regulator of IL-6 transcription, demonstrating that its regulatory outcome is fundamentally determined by the specific promoter architecture it occupies. Our results reveal a complex regulatory landscape where FOXO3 acts as a potent activator when binding to proximal elements, yet transitions into a repressor when interacting with more distal regions of the IL-6 promoter. This context-dependent bimodality provides a novel mechanism for the fine-tuning of inflammatory responses. In conclusion, our study highlights the IL-6 promoter as a sophisticated regulatory platform where FOXO3 serves as a molecular switch, dictating the magnitude and direction of IL-6 transcription in a promoter-dependent manner, particularly relevant during mycobacterial infection. Future research should focus on identifying the specific co-factors recruited to the 2.0 kb distal region in both infected and non-infected cells, and characterizing the post-translational modifications of FOXO3 that dictate its site-specific activity. Furthermore, investigating the interplay between FOXO3 and other BCG-activated signaling pathways like NF-κB, which is also involved in IL-6 regulation (33) will be essential for a comprehensive understanding of IL-6 control during mycobacterial challenge.

Understanding these nuances will be critical for developing targeted interventions that can precisely modulate IL-6 levels and optimize host immune responses in infectious diseases.

**Figure 8:**
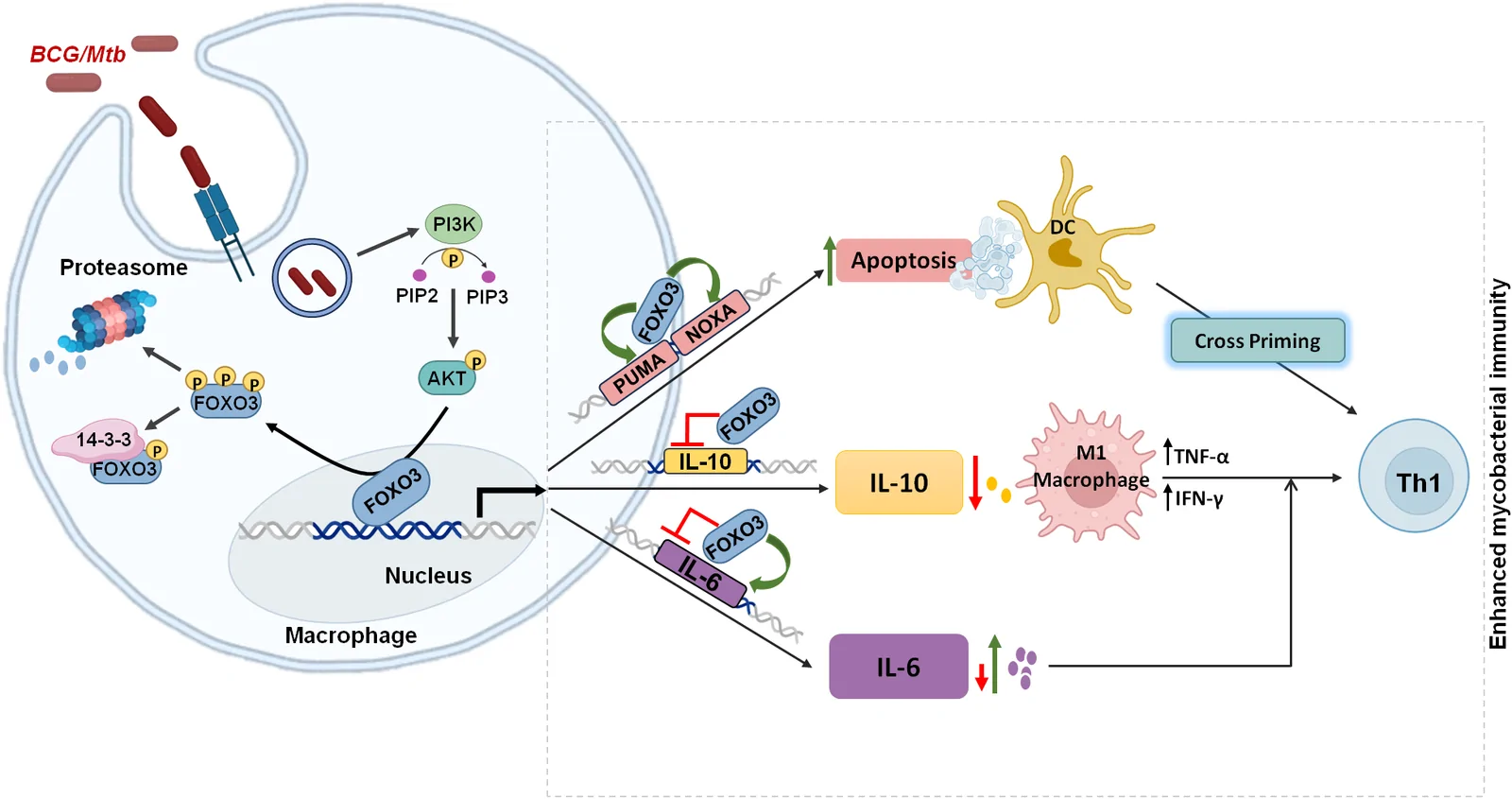
FOXO3: a central regulator of the immune response against mycobacterial infection. FOXO3 serves as a critical signaling hub orchestrating a protective immune response against mycobacterial infection. *Mtb* often subverts host resistance by predominantly activating the pro-survival PI3K/Akt pathway. Activated Akt phosphorylates the transcription factor FOXO3 at three key residues (Thr32, Ser253, and Ser315), facilitating its translocation to the cytosol where it is sequestered by chaperone protein 14-3–3 and/or targeted for proteasomal degradation. Conversely, inhibition of the PI3K/Akt pathway leads to the activation of FOXO3 and its translocation to the nucleus, where it induces apoptosis through the upregulation of pro-apoptotic mediators such as PUMA and NOXA. Furthermore, nuclear FOXO3 directly binds to the proximal promoter of IL-10, inhibiting its secretion. While also inducing IL-6 expression, this finely tuned regulation of IL-6 by FOXO3 ultimately strengthens the M1/Th1 immune response, leading to effective control of the infection. Inhibition of IL-10 signaling is crucial for restoring the host’s anti-mycobacterial capacity and promoting the polarization of macrophages toward a pro-inflammatory M1 phenotype, which is essential for shaping an effective T-helper 1 (Th1) adaptive response against *Mtb* pathogenesis. FOXO3-mediated apoptosis generates apoptotic bodies charged with mycobacterial antigens. These apoptotic bodies are subsequently ingested by bystander dendritic cells (DCs), facilitating effective cross-priming of T cells. The positive regulation of IL-6 by FOXO3 further reinforces the M1/Th1 immune response, contributing to robust infection control. Importantly, FOXO3 also exerts negative regulation on a distant fragment of the IL-6 promoter, thereby restraining excessive IL-6 production that could otherwise exacerbate detrimental host inflammatory responses.

In summary, this study and our previous works indicate that activation of FOXO3 in infected macrophages confers multifaceted protection against tuberculosis: (i) Induction of apoptosis of infected macrophages, thereby disrupting the intracellular replication of *Mtb* and enhancing the cross priming presentation to CD8 T cells. (ii) Inhibition of IL-10 secretion, increasing the host’s antimicrobial responses through directing the immune response toward an M1/Th1 phenotype. (iii) Finely tuning IL-6 expression to boost the adaptive immune response against *Mtb* while preventing excessive inflammation (**Figure 8**).

## Data Availability

The original contributions presented in the study are included in the article/**Supplementary Material**. Further inquiries can be directed to the corresponding author.
