# Supplementary material for: Forkhead box O3 transcription factor fine-tunes IL-6 expression in mycobacteria-infected macrophages
Source: Front Immunol. 2026 Aug 17;17:1888327. doi: 10.3389/fimmu.2026.1888327 (PMC13526617; doi:10.3389/fimmu.2026.1888327)
Supplement: Supplementary file 1 [file Table1.docx]

**Table S1.** Sequences of the used primers to quantify human gene expression by RT-qPCR.

| Gene | **Primer** | **Sequence (5'-3')** |
| --- | --- | --- |
| GAPDH | Forward  Reverse | GGATTTGGTCGTATTGGG  GGAAGATGGTGATGGGATT |
| *β-*ACTIN: | Forward  Reverse | CACCAACTGGGACGACAT  ACAGCCTGGATAGCAACG |
| FOXO3 | Forward  Reverse | AAATGAAAGCTCACTCTGGATTCC TGTGCAATTCCTATG CAATC |
| IL-6 | Forward  Reverse | AATCATCACTGGTCTTTTGGAG  GGTTATTGCATCTAGATTCTTTGC |

**Table S2.** Oligonucléotide primers used for cloning the different IL-6 promoter constructs.

| IL-6 promoter fragment | Primer sequence (5'-3') |
| --- | --- |
| 600bp (V1) | **F** GCAGCTAGCTGAACACGAACTTCCTATTT  **R** GAGAAGCTTCTCCAGTCTCCAGAGACCCA |
| 900bp (V2) | **F** GTGAGATCTCACAGAAGAACTCAGATGACTG  **R** GGAAAGCTTTCTTTGGAATCTTCTCCTGGG |
| 1.5kb (V3) | **F** GATGTCGACACAACCTAGTCTAGAGCCCA  **R** GGAAAGCTTTCTTTGGAATCTTCTCCTGGG |
| 2kb (V4) | **F** GAGGTCGACCTGCATGCCCTGATGTCCTATT  **R** GGAAAGCTTTCTTTGGAATCTTCTCCTGGG |

**Table S3.** Sequences of the used primers to disrupt FOXO3 binding motifs on the human IL-6 promoter constructs.

| Position on IL-6 promoter | Primer sequence (5'-3') |
| --- | --- |
| -786 | **F** GTTCAGAACATCTTTGGTT**T**CAAATACAAATTAACTGG  **R** CCAGTTAATTTGTATTTGA**A**ACCAAAGATGTTCTGAAC |
| -1331 | **F** AGAGGGAGAGGGAGCGAT**A**CAAACTCTGCAAGATGCCA  **R** TGGCATCTTGCAGAGTTTG**T**ATCGCTCCCTCTCCCTCT |
| -1631 | **F** GTAACACCATGTTTGGTA**A**AGTGTTTTGGTGTTGTGC  **R** GCACAACACCAAAACACT**T**TACCAAACATGGTGTTAC |
